# Assessment of aortic diameter in Marfan patients: intraindividual comparison of 3D-Dixon and 2D-SSFP magnetic resonance imaging

**DOI:** 10.1007/s00330-022-09162-y

**Published:** 2022-10-21

**Authors:** Felicia Wright, Malte Warncke, Martin Sinn, Inka Ristow, Alexander Lenz, Christoph Riedel, Bjoern P. Schoennagel, Shuo Zhang, Michael G. Kaul, Gerhard Adam, Yskert von Kodolitsch, Susanne Sehner, Peter Bannas

**Affiliations:** 1 Department of Diagnostic and Interventional Radiology and Nuclear Medicine, University Medical Center Hamburg-Eppendorf, Martinistraße 52, 20246 Hamburg, Germany; 2 Philips GmbH Market DACH, Hamburg, Germany; 3 Department of Vascular Medicine, University Heart and Vascular Center Hamburg, Hamburg, Germany; 4 Institute of Medical Biometry and Epidemiology, University Medical Center Hamburg-Eppendorf, Hamburg, Germany

**Keywords:** Marfan syndrome, Magnetic resonance angiography, Artifacts, Echocardiography, Thoracic aorta

## Abstract

**Objectives:**

To compare the accuracy and precision of 3D-Dixon and 2D-SSFP MR-imaging for assessment of aortic diameter in Marfan patients.

**Methods:**

This prospective single-center study investigated respiratory-gated 3D-Dixon and breath-hold 2D-SSFP non-contrast MR-imaging at 3 T in 47 Marfan patients (36.0 ± 13.2 years, 28♀,19♂). Two radiologists performed individual diameter measurements at five levels of the thoracic aorta and evaluated image quality on a four-grade scale (1 = poor, 4 = excellent) and artifacts (1 = severe, 4 = none). Aortic root diameters acquired by echocardiography served as a reference standard. Intraclass correlation coefficient, Bland-Altman analyses, F-test, t-test, and regression analyses were used to assess agreement between observers and methods.

**Results:**

Greatest aortic diameters were observed at the level of the sinuses of Valsalva (SOV) for 3D-Dixon (38.2 ± 6.8 mm) and 2D-SSFP (38.3 ± 7.1 mm) (*p* = 0.53). Intra- and interobserver correlation of diameter measurements was excellent at all aortic levels for both 3D-Dixon (*r* = 0.94–0.99 and *r* = 0.94–0.98) and 2D-SSFP (*r* = 0.96–1.00 and *r* = 0.95–0.99). 3D-Dixon-derived and 2D-SSFP-derived diameter measurements at the level of the SOV revealed a strong correlation with echocardiographic measurements (*r* = 0.92, *p* < 0.001 and *r* = 0.93, *p* < 0.001, respectively). The estimated mean image quality at the level of SOV was higher for 2D-SSFP compared to that for 3D-Dixon (3.3 (95%-CI: 3.1–3.5) vs. 2.9 (95%-CI: 2.7–3.1)) (*p* < 0.001). Imaging artifacts were less at all aortic levels for 3D-Dixon compared to 2D-SSFP (3.4–3.8 vs. 2.8–3.1) (all *p* < 0.002).

**Conclusion:**

Respiratory-gated 3D-Dixon and breath-hold 2D-SSFP MR-imaging provide accurate and precise aortic diameter measurements. We recommend 3D-Dixon imaging for monitoring of aortic diameter in Marfan patients due to fewer imaging artifacts and the possibility of orthogonal multiplanar reformations of the aortic root.

**Key Points:**

*• Respiratory-gated 3D-Dixon and breath-hold 2D-SSFP imaging provide accurate and precise aortic diameter measurements in patients suffering from Marfan syndrome.*

*• Imaging artifacts are stronger in 2D-SFFP imaging than in 3D-Dixon imaging.*

*• We recommend 3D-Dixon imaging for monitoring of aortic diameter in Marfan patients due to fewer imaging artifacts and the possibility of orthogonal multiplanar reformations.*

## Introduction

Marfan syndrome is an autosomal-dominant inherited genetic disorder of the connective tissue with a prevalence of one in 5,000–10,000 individuals [1, 2]. General insufficiency of the connective tissue is caused by mutations in the FBN1 gene encoding the protein Fibrillin [3]. This disease affects different parts of the human body, including the heart and blood vessels [4, 5]. The most common cardiovascular complication is progressive dilatation of the aortic root [3, 6, 7]. The main cause of death in undetected Marfan syndrome is the dissection of the dilatated aortic root [3, 6–8]. The survival of Marfan patients has significantly improved due to prophylactic medication and elective repair of the aortic root [3, 9, 10].

Life-long annual aortic imaging is mandatory to determine the indication and timepoint of aortic root replacement in Marfan patients [3, 10]. Elective surgery is indicated in the case of aortic root dilatation of ≥ 5 mm/year or an absolute aortic diameter of ≥ 50 mm [6, 11–13]. Accurate cross-sectional, and non-invasive radiation-free imaging techniques are needed for the life-long annual aortic imaging in Marfan patients.

Magnetic resonance imaging (MRI) allows for accurate, operator-independent, and radiation-free assessment of aortic diameters [14, 15]. Previous studies revealed an excellent quality of breath-hold 2D balanced steady-state with free precession (2D-SSFP) imaging with coverage of the entire thoracic aorta [16]. Non-contrast 2D-SSFP MR imaging avoids the risk of adverse contrast agent reactions, nephrogenic systemic fibrosis, or cerebral gadolinium deposition, which may occur when performing contrast-enhanced MR-angiography [11, 13]. Recent studies confirmed that 2D-SSFP imaging allows for accurate monitoring of aortic root diameters in pre- and postoperative Marfan patients [8, 17].

However, 2D acquisition precludes secondary multiplanar reformations for exact orthogonal measurements, while non-orthogonal angulation may lead to overestimation of aortic root diameters and/or growth rates [6] and potentially results in the wrong clinical management of Marfan patients.

On the other hand, it is known that balanced SSFP techniques suffer from high sensitivity to off-resonance effects caused by B0 inhomogeneities, particularly at higher field strengths (≥ 3 T) [6]. The resulting banding and pulsation artifacts, high background signals, signal loss, and insufficient fat suppression often lead to decreased image quality in SSFP images, which is even worse in the case of post-surgical Marfan patients having metallic implants.

In recent years, various imaging techniques based on Dixon water-fat separation and magnetization preparation, e.g. T2-prep [18, 19], have been demonstrated as being effective for delineation of cardiothoracic vasculatures at 3 T [13, 20]. In addition, respiratory gating and cardiac end-diastolic triggering can be added for improved motion robustness in imaging large thoracic vessels [21, 22].

The purpose of our single-center prospective study was to compare the accuracy and precision of the newly developed respiratory-gated 3D-Dixon sequence and the established breath-hold 2D-SSFP imaging sequence for the assessment of aortic diameter in Marfan patients.

## Material and methods

### Study population

This prospective study was approved by the institutional review board and all subjects provided written informed consent.

Forty-seven adult patients (19 male, 28 female; age range 18–71 years, mean 36.0 ± 13.2 years) with confirmed Marfan syndrome prior to aortic surgery were consecutively included between May 2019 and August 2020. Inclusion criteria comprised Marfan diagnosis according to the latest Ghent nosology and verification of FBN1 mutation [11]. Exclusion criteria were previous aortic surgery and known contraindications for MRI [7].

All patients underwent both MR imaging and routine transthoracic echocardiography on the same day.

### MR imaging

MR imaging was performed using a 3-T clinical whole-body MRI system equipped with a 32-channel arrayed coil (Philips Ingenia, Philips Medical Systems). MR-compatible ECG electrodes were placed in a standardized manner for cardiac triggering. Scout images in axial, coronal, and sagittal orientation covering the thorax were performed at the beginning of every examination.

#### Free-breathing 3D-Dixon imaging

3D dual-echo gradient-echo Dixon (3D-Dixon) [19, 21] images were acquired in para-sagittal orientation aligned with the aortic arch during free breathing. In line with current guidelines for the diagnosis and management of patients with the thoracic aortic disease [23] and previous studies [8, 17] standardized para-sagittal *“candy cane”* views of the complete thoracic aorta, planned on transversal scout images and orientated centrally through the aortic arch, were acquired.

Both respiratory navigator gating and end-diastolic cardiac triggering were applied to minimize motion artifacts. A 5-mm respiratory gating window was used for tracking a consistent end-expiratory phase throughout data acquisition. Main imaging parameters were as follows: TR/TE, 3.8/1.32 ms; flip angle, 15°; FOV 400 mm (FH) x 507 mm (AP) x 150 mm (RL); matrix, 268 x 337 x 150; number of slices 150; acquired voxel size: 1.5 mm x 1.5 mm x 2.0 mm; compressed sense factor: 6.5. Acquisition time was 8–10 min, depending on the individual heart rate and respiratory frequency.

#### Breath-hold 2D steady-state free precession imaging

2D balanced steady-state free precession (SSFP) was acquired during breath-holding in para-sagittal orientation aligned with the aortic arch as described above. The acquisition was gated to the end-diastolic phase of the cardiac cycle for the minimization of motion artifacts. Main imaging parameters were as follows: TR/TE, 2.3/0.89 ms; flip angle, 60°; FOV 280 mm (FH) x 362 mm (AP) x 113 mm (RL); matrix, 216 x 282 x 27; number of slices 27; acquired voxel size: 1.3 mm x 1.3 mm x 9 mm with an overlap of 5 mm; SENSE factor, 4. The acquisition time for each stack was 11–16 s, depending on the individual heart rate and breath-holding capability.

### MR image evaluation

Anonymised free-breathing 3D-Dixon images and breath-hold 2D-SSFP images were presented in random order to two radiologists, with 4 years (F.W.) and 5 years (M.W.) of experience in cardiovascular imaging. All images were interpreted on state-of-the-art RIS/PACS workstations (Centricity^TM^ RIS-i 4.2 Plus, General Electric Company).

**Fig. 1:**
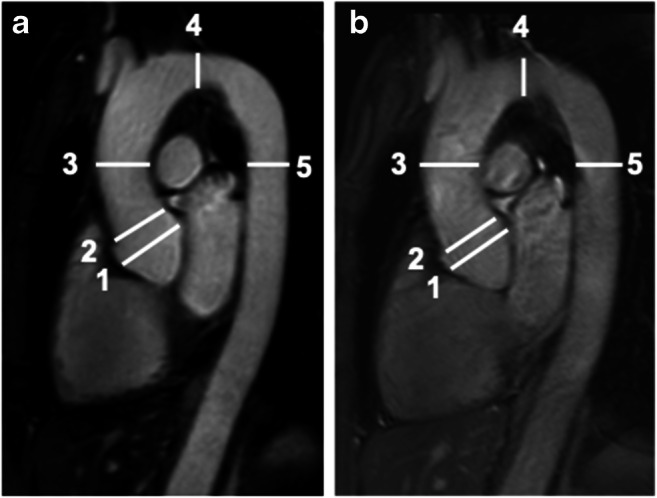
Comparison of 3D-Dixon and 2D-SSFP MR-imaging of the thoracic aorta with indicated measurement levels. Para-sagittal 3D-Dixon (**a**) and 2D-SSFP MR-imaging (**b**) in a 36-year-old woman with confirmed Marfan syndrome. White lines indicate the five aortic measurement levels for diameter as well as image quality and artifact estimation: (1) sinuses of Valsalva, (2) sinotubular junction, (3) ascending aorta, (4) mid aortic arch, and (5) descending aorta

Aortic diameter measurements and image quality assessments were performed at five predefined levels: sinuses of Valsalva (SOV), sinotubular junction, ascending aorta at the level of the pulmonary trunk, mid aortic arch between the branching of left carotid and left subclavian artery, and descending aorta at the level of the pulmonary trunk as illustrated in Fig. 1 [12].

#### Aortic diameter measurements

Both readers performed individual inner aortic diameter measurements at all predefined levels perpendicular to the blood-filled lumen on identically orientated para-sagittal 3D-Dixon and 2D-SSFP source images. The readers were free to choose appropriate slices displaying the maximal profile of the aorta from the stacks of parasagittal images. No secondary multiplanar reformations (MPR) were used for the comparison of the two imaging techniques. Using identically oriented para-sagittal source images avoided user influence introduced by individually performed MPRs and allowed for assessment of only the differences that are attributed to the image quality (i.e., delineation of the vessel wall) of the two different imaging techniques [12, 24].

Aortic diameters were measured three times: reader 1 performed two measurements for the assessment of intraobserver agreement, with an interval of 6 weeks between both measurements. Reader 2 performed a third measurement for the assessment of interobserver agreement. 3D-Dixon-based and 2D-SSFP-based diameter measurements were performed in two different reading sessions with a 3-week time interval between assessments of the same case to avoid recall bias.

#### Qualitative MR image quality and artifact evaluation

The image quality of both 3D-Dixon and 2D-SSFP images was assessed by both readers individually at all aortic levels regarding the sharp anatomic delineation of the inner aortic vessel wall on a four-point Likert scale [6, 12]:

- 1 = poor image quality, poorly defined anatomic details, poor diagnostic confidence
- 2 = reduced image quality, limitations in anatomic detail, impairment of diagnostic confidence
- 3 = good image quality, clear anatomic details, slightly blurred delineation of the aortic inner vessel wall, no impairment of diagnostic confidence
- 4 = excellent image quality, distinct anatomic details, full diagnostic confidence

Artifacts of both 3D-Dixon and 2D-SSFP images were assessed by both readers individually at all aortic levels on a four-point Likert scale [6, 7, 10].

- 1 = severe artifacts, non-diagnostic image
- 2 = moderate artifacts, degrading diagnostic content
- 3 = minor artifacts, not interfering with diagnostic content
- 4 = no artifacts

#### Quantitative MR image evaluation

An in-house developed plugin (QMapIt) for ImageJ [25] was used to semi-automatically assess the vessel wall sharpness of both 3D-Dixon and 2D-SSFP imaging. First, a perpendicular line was drawn through the aortic lumen and the adjacent aortic walls at the level of the sinuses of Valsalva. The mean up- and down-slopes between two turning points of the parabolic-shaped signal intensity curves were automatically calculated, representing the sharpness of the aortic vessel wall [6, 26].

### Echocardiography

All included Marfan patients underwent 2D transthoracic echocardiography as part of their routine screening at our University Heart and Vascular Center. 2D-transthoracic echocardiography was performed according to current guidelines [27] with a standard ultrasound system (EPIQ CVx, Philips). End-diastolic aortic root diameters were determined by experienced cardiologists using the leading-edge method in the parasternal long axis view at the level of the SOV [28]. Diameters as determined by echocardiography served as a reference standard and were statistically compared to 3D-Dixon-based and 2D-SSFP-based diameter measurements.

### Statistical analysis

Means and standard deviations were calculated for 3D-Dixon-derived and 2D-SSFP-derived aortic diameters. Data is shown as mean +/- SD unless stated otherwise.

3D-Dixon-derived diameters and 2D-SSFP-derived diameters were compared with each other and with echocardiographic measurements using Bland-Altman analyses. Pearson´s correlation (stated as cursive “*r*”) was used to determine significant differences between aortic diameters derived from 3D-Dixon and 2D-SSFP imaging as well as echocardiography.

Intraclass correlation coefficients (stated as standard “*r*”) were calculated to assess intra- and interobserver correlation of 3D-Dixon-based and 2D-SSFP-based diameter measurements. Correlation coefficients > 0.8 indicated strong correlation and correlation coefficients ≥ 0.94 indicated excellent correlation.

Bland-Altman analysis was used to assess intra- and interobserver agreement between 3D-Dixon-based and 2D-SSFP-based aortic diameter measurements. A two-sided paired t-test was performed for comparison of mean differences (bias) and *F*-test for comparison of variances.

Effects of 3D-Dixon and 2D-SSFP imaging on the evaluation of image quality and artifacts with respect to the used Likert scale were estimated with a multilevel mixed-effect linear regression. Additionally, a multilevel mixed-effect ordered logistic regression was performed to account for the ordinal structure of the Likert scale. Both approaches account for the dependency structure of the data, resulting from the repeated measurement at the five predefined levels with both imaging techniques in each patient and the assessment of each image by the readers, by modelling a random intercept for patients and readers.

In both modelling approaches, an interaction term between technique and level was included to compare the imaging procedures with respect to 3D-Dixon and 2D-SSFP as a function of the level. In the case of a non-significant interaction term, only the two main effects would remain in the model. This decision was made using the likelihood ratio test for model comparison. Both models showed a significant difference between 3D-Dixon and 2D-SSFP, which, however, depends on the level assessed (*p* value for level by imaging technique interaction < 0.001 for all).

The results of image quality and artifact rating were reported as estimated marginal means with corresponding 95% confidence intervals (95%-CIs) for the linear and estimated probabilities for the ordered logistic regression.

Means and standard deviations were calculated for 3D-Dixon-derived and 2D-SSFP-derived up- and down-slopes of signal intensity curves of vessel walls. A two-sided paired t-test was performed for the comparison of mean differences.

*p* values < 0.05 were considered statistically significant. Statistical analyses were performed using Excel 14.7.2 (Microsoft Cooperation) and SPSS 26 (IBM).

## Results

All 47 3D-Dixon and 2D-SSFP scans were deemed with diagnostic image quality and allowed for aortic measurements at all levels. None of the patients had an aortic dissection or mural thrombi.

### Intraobserver agreement of aortic diameter measurements

*Intraobserver correlation* was excellent at all aortic levels for both 3D-Dixon (*r* = 0.94–0.99) and 2D-SSFP imaging (*r* = 0.96–1.00) (Table 1).

**Table 1:** Intraobserver variance of measured aortic diameters obtained by 3D-Dixon and 2D-SSFP MR-imaging as described by Bland and Altman

Intraobserver variance					
	Sinuses of Valsalva	Sinotubular junction	Ascending aorta	Mid aortic arch	Descending aorta
**3D-Dixon**					
Mean difference (mm)	−0.09	−0.19	−0.23	−0.13	−0.26
Limit of agreements (mm)	−2.60 to 2.43	−4.70 to 4.32	−2.98 to 2.52	−2.64 to 2.38	−2.55 to 2.04
Standard deviation (mm)	1.28	2.30	1.40	1.28	1.17
Variance (mm^2^)	1.64	5.29	1.97	1.64	1.37
Intraclass correlation coefficient (*r*)	0.99	0.94	0.98	0.96	0.97
**2D-SSFP**					
Mean difference (mm)	−0.13	−0.02	0.02	−0.15	−0.02
Limit of agreements (mm)	−1.74 to 1.49	−3.97 to 3.93	−2.20 to 2.24	−2.57 to 2.27	−2.16 to 2.12
Standard deviation (mm)	0.82	2.02	1.13	1.23	1.09
Variance (mm^2^)	0.68	4.07	1.28	1.52	1.20
Intraclass correlation coefficient (*r*)	1.00	0.96	0.99	0.96	0.98
*p* value (*t* test)	0.81	0.68	0.54	0.93	0.31
*p* value (*F* test)	**< 0.001**	0.19	0.08	0.40	0.32

*T*-test was performed for comparison of mean differences and *F*-test for comparison of variances. Significant differences are in boldface (significant at *p* < 0.05)

*Intraobserver bias* revealed no statistically significant difference between 3D-Dixon and 2D-SSFP imaging at all defined aortic levels (all *p* > 0.31) (Table 1).

**Fig. 2:**
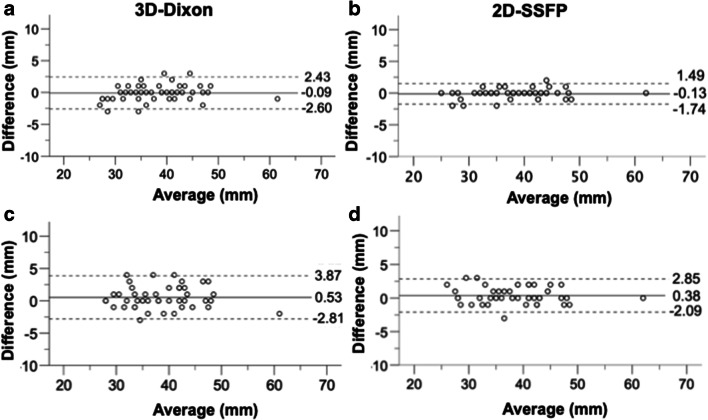
Intra- and interobserver agreement of 3D-Dixon and 2D-SSFP diameter measurements at the sinuses of Valsalva. Bland-Altman analyses of intraobserver (**a, b**) and interobserver agreement (**c**, **d**) demonstrate significantly greater variances of 3D-Dixon as compared to 2D-SSFP measurements (*p* < 0.001 and *p* = 0.02, respectively). *Dotted lines* indicate limits of agreement and middle *solid line* indicates the mean bias of diameter measurements

*Intraobserver variance* was significantly greater for the SOV for 3D-Dixon imaging (limits of agreement: −2.60 to 2.43 mm) than for 2D-SSFP (limits of agreement: −1.74 to 1.49 mm) (*p* < 0.001) (Fig. 2a, b). There was no statistically significant difference regarding intraobserver variances at all other aortic levels (all *p* ≥ 0.08) (Table 1).

### Interobserver agreement of aortic diameter measurements

*Interobserver correlation* was excellent at all aortic levels for both 3D-Dixon (*r* = 0.94-0.98) and 2D-SSFP (*r* = 0.95–0.99) (Table 2).

**Table 2:** Interobserver variance of the measured aortic diameters obtained by 3D-Dixon and 2D-SSFP MR-imaging as described by Bland and Altman

Interobserver variance					
	Sinuses of Valsalva	Sinutubular junction	Ascending aorta	Mid aortic arch	Descending aorta
**3D-Dixon**					
Mean difference (mm)	0.53	0.57	1.00	0.40	0.15
Limit of agreements (mm)	−2.81 to 3.87	−1.97 to 3.11	−2.19 to 4.19	−2.33 to 3.14	−3.04 to 3.34
Standard deviation (mm)	1.70	1.30	1.63	1.39	1.63
Variance (mm^2^)	2.91	1.69	2.65	1.94	2.65
Intraclass correlation coefficient (*r*)	0.98	0.98	0.98	0.94	0.94
**2D-SSFP**					
Mean difference (mm)	0.38	0.13	0.87	−0.04	0.47
Limit of agreements (mm)	− 2.09 to 2.85	−2.92 to 3.18	2.20 to 3.95	−2.81 to 2.73	−2.59 to 3.52
Standard deviation (mm)	1.26	1.56	1.57	1.41	1.56
Variance (mm^2^)	1.59	2.42	2.46	2.00	2.43
Intraclass correlation coefficient (*r*)	0.99	0.98	0.98	0.95	0.95
*p* value (*t* test)	0.55	0.88	0.66	0.15	0.21
*p* value (*F* test)	**0.02**	0.11	0.40	0.46	0.38

*T*-test was performed for comparison of mean differences and *F*-test for comparison of variances. Significant differences are in boldface (significant at *p* < 0.05)

*Interobserver bias* was without a statistically significant difference between 3D-Dixon and 2D-SSFP imaging at all aortic levels (all *p* ≥ 0.15) (Table 2).

*Interobserver variance* was significantly greater for the SOV for 3D-Dixon (limits of agreement: −2.81 to 3.87 mm) than for 2D-SSFP imaging (limits of agreement: −2.09 to 2.85 mm) (*p* = 0.02) (Fig. 2c, d). There was no statistically significant difference in interobserver variances at all other aortic levels (all *p* ≥ 0.11) (Table 2).

### Comparison of 3D-Dixon- and 2D-SSFP-derived aortic diameter measurements

**Fig. 3:**
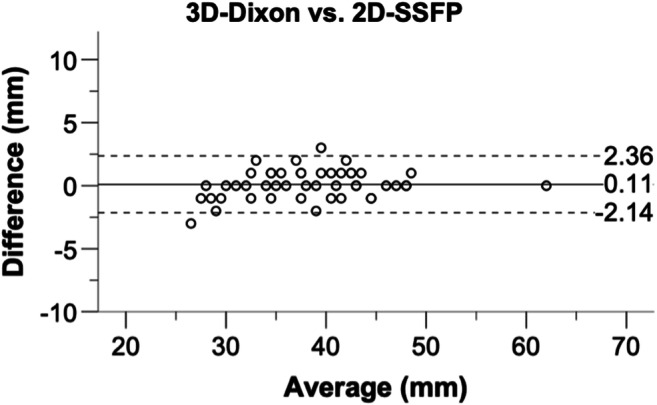
Bland-Altman comparison of 3D-Dixon vs. 2D-SSFP diameter measurements at the sinuses of Valsalva. The plot reveals a non-significant mean difference of 0.1 ± 2.3 mm between 3D-Dixon and 2D-SSFP-derived measurements (*p* = 0.53). *Dotted lines* indicate limits of agreement and the middle *solid line* indicates mean bias of diameter measurements

The greatest aortic diameters were observed at the level of the SOV. There was no significant difference in mean diameters between 3D-Dixon imaging (38.2 ± 6.8 mm) and 2D-SSFP imaging (38.3 ± 7.1 mm) (*p* = 0.53) (Fig. 3, Table 3). There was also no significant difference between 3D-Dixon- and 2D-SSFP-derived aortic diameters at the level of the sinotubular junction, ascending aorta, and aortic arch (all *p* ≥ 0.16) (Table 3). The only significant difference in aortic diameters was observed at the level of the descending aorta between 3D-Dixon imaging (22.6 ± 3.2 mm) and 2D-SSFP imaging (20.9 ± 3.6 mm) (*p* = 0.001) (Table 3).

**Table 3:** Comparison of the aortic diameters as determined by 2D-SSFP and 3D-Dixon MR-imaging as described by Bland and Altman

3D-Dixon vs. 2D-SSFP	Sinuses of Valsalva	Sinotubular junction	Ascending aorta	Mid aortic arch	Descending aorta
Mean diameter 3D-Dixon (mm)	38.2 ± 6.8	28.1 ± 5.5	27.4 ± 5.4	22.0 ± 3.2	22.6 ± 3.2
Mean diameter 2D-SSFP (mm)	38.3 ± 7.1	28.2 ± 5.0	27.0 ± 5.1	22.0 ± 3.3	20.9 ± 3.6
Mean difference (mm)	0.1	0.2	−0.4	0.1	−1.7
Limit of agreements (mm)	−2.1 to 2.4	−4.9 to 5.3	−4.4 to 3.6	−2.7 to 2.9	−4.9 to 1.5
Standard deviation (mm)	1.1	2.6	2.0	1.4	1.6
Variance (mm^2^)	1.3	6.8	4.2	2.1	2.7
*p* value (*t* test)	0.53	0.66	0.16	0.69	**0.001**
Pearson´s correlation (*r*)	0.99	0.88	0.93	0.9	0.89

Pearson´s correlation coefficient (*r*) between different imaging techniques is given. *T*-test was performed for comparison of mean differences. Significant differences are in boldface (significant at *p* < 0.05)

Pearson´s correlation revealed a strong correlation of diameters obtained by 3D-Dixon and 2D-SSFP imaging at all aortic levels (*r* = 0.88–0.99) (Table 3).

### Comparison of MRI-derived aortic measurements and echocardiography

Pearson´s correlation analysis revealed strong correlation of SOV diameter measurements derived from both 3D-Dixon imaging (*r* = 0.92, *p* < 0.001), 2D-SSFP imaging (*r* = 0.93, *p* < 0.001) and echocardiography.

**Fig. 4:**
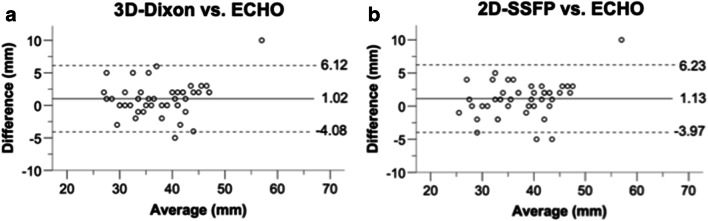
Bland-Altman analyses of 3D-Dixon and 2D-SSFP measurements in comparison to echocardiographic measurements at the sinuses of Valsalva. Bland-Altman plots demonstrate statistically significant mean biases of 1.0 mm (limits of agreement: −4.08 to 6.11 mm) for 3D-Dixon (**a**) and of 1.1 mm (limits of agreement: −3.97 to 6.23 mm) for 2D-SSFP measurements (**b**) when compared to echocardiographic diameter measurements (*p* = 0.010 and *p* = 0.005, respectively). *Dotted lines* indicate limits of agreement and the middle *solid line* indicates mean bias of diameter measurements

SOV diameter measurements derived from both 3D-Dixon imaging (38.2 ± 6.8 mm) and 2D-SSFP imaging (38.3 ± 7.1 mm) were significantly larger (*p* = 0.01 and *p* = 0.005, respectively) than echocardiographic measurements (37.2 ± 6.2 mm). The Bland-Altman analysis demonstrated a mean bias of 1.0 mm (limits of agreement: −4.08 to 6.11 mm) for 3D-Dixon and 1.1 mm (limits of agreement: −3.97 to 6.23 mm) for 2D-SSFP imaging when compared with echocardiographic diameter measurement at the SOV (Fig. 4).

### Image quality and artifact scoring of 3D-Dixon and 2D-SFFP imaging

**Fig. 5:**
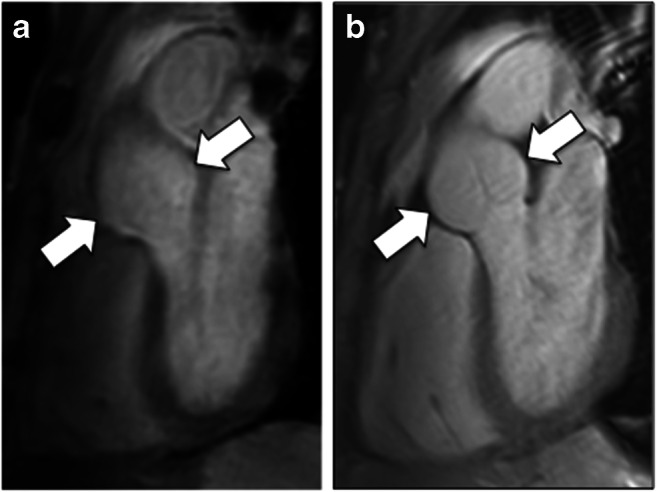
Comparison of image quality and image artifacts of respiratory-gated 3D-Dixon and breath-hold 2D-SSFP imaging. Parasagittal respiratory-gated 3D-Dixon imaging (**a**) resulted in a blurred delineation of the sinuses of Valsalva (arrows), while breath-hold 2D-SSFP imaging (**b**) resulted in sharper delineation of the sinuses of Valsalva (arrows) in this 43-year-old male patient with confirmed Marfan syndrome. Both readers rated the image quality at the sinuses of Valsalva as an average of 3.5 points (= good) for 3D-Dixon and 4 points (= excellent) for 2D-SSFP. Both readers rated the image artifacts as an average of 4 points (excellent) for 3D-Dixon and 3.5 points (= good) for 2D-SSFP. Both 3D-Dixon-derived and 2D-SSFP-derived measurements resulted in a maximum aortic diameter of 34 mm at the level of the sinuses of Valsalva

**Fig. 6:**
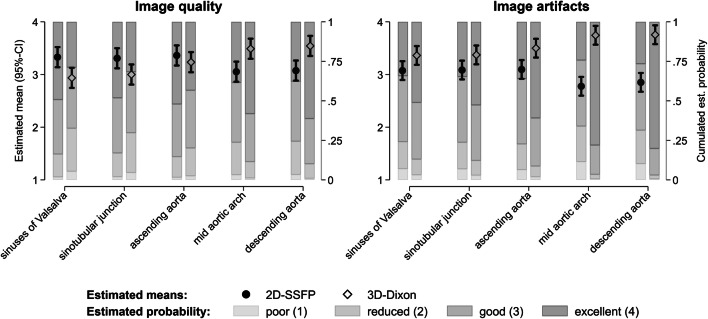
Qualitative analyses of image quality and image artifacts of respiratory-gated 3D-Dixon and breath-hold 2D-SSFP imaging. Results of image quality and artifact rating are reported for 2D-SSFP (*black circles*) and 3D-Dixon (*white diamonds*) as estimated marginal means with corresponding 95% confidence intervals (95%-CIs) and cumulated estimated probabilities. Four-point scales were used for image quality rating (1 = poor, 4 = excellent) and artifact rating (1 = severe, 4 = none)

Image quality scoring revealed good to the excellent delineation of the aortic inner vessel wall at all aortic levels for both 3D-Dixon and 2D-SSFP acquisitions. However, respiratory-gated 3D-Dixon imaging resulted in a slightly blurred delineation of the aortic inner vessel wall, while breath-hold 2D-SSFP imaging resulted in the sharper delineation of the aortic inner vessel wall (Fig. 5). This resulted in significantly higher image quality scores for 2D-SSFP compared with 3D-Dixon imaging at the aortic root (sinuses of Valsalva, sinotubular junction) (both *p* ≤ 0.001) (Table 4, Fig. 6).

**Table 4:** Estimated marginal means of image quality and image artifacts scores of both readers for 3D-Dixon and 2D-SSFP MR-imaging

Estimated means (95%-CI)	Sinuses of Valsalva	Sinotubular junction	Ascending aorta	Mid aortic arch	Descending aorta
**Image quality**					
3D-Dixon	2.9	3.0	3.2	3.5	3.5
	(2.7–3.1)	(2.8–3.2)	(3.0–3.4)	(3.3–3.7)	(3.4–3.7)
2D-SSFP	3.3	3.3	3.4	3.1	3.1
	(3.1–3.5)	(3.1–3.5)	(3.2–3.6)	(2.9–3.2)	(2.9–3.3)
Model-based *p *value	< 0.001	0.001	0.162	< 0.001	< 0.001
for pairwise comparison					
**Presence of artifacts**					
3D-Dixon	3.4	3.4	3.5	3.7	3.8
	(3.2–3.5)	(3.2–3.6)	(3.3–3.7)	(3.6–3.9)	(3.6–3.9)
2D-SSFP	3.1	3.1	3.1	2.8	2.9
	(2.9–3.3)	(2.9–3.3)	(2.9–3.3)	(2.6 – 3.0)	(2.7–3)
Model-based *p* value	0.002	0.002	< 0.001	< 0.001	< 0.001
for pairwise comparison					

Four-point scales were used for image quality rating (1 = poor, 4 = excellent) and artifact rating (1 = severe, 4 = none). Results of image quality and artifact rating are reported as estimated marginal means with corresponding 95% confidence intervals (95%-CIs)

Artifact scoring revealed statistically significant stronger artifacts at all aortic levels for 2D-SSFP imaging than for 3D-Dixon imaging (all *p* < 0.002) (Table 4, Fig. 6).

### Quantitative assessment of vessel wall sharpness of 3D-Dixon and 2D-SFFP imaging

Quantitative analyses of the sharpness of the vessel wall at the level of the sinuses of Valsalva were higher for 2D-SSPP than for 3D-Dixon acquisitions at both the anteriorly (up-slope) and posteriorly (down-slope) orientated vessel wall. Significantly steeper up-slopes (74 ± 34 vs. 32 ± 16, *p* < 0.0001) and down-slopes (76 ± 28 vs. 31 ± 19, *p* < 0.0001) of the signal intensity curves, representing the transition of the aortic lumen to both aortic walls, were observed levels at 2D-SSFP as compared with 3D-Dixon imaging.

## Discussion

This prospective single-center study demonstrated high accuracy for both respiratory-gated 3D-Dixon and breath-hold 2D-SSFP MR-imaging for assessment of aortic diameter in Marfan patients. Breath-hold 2D-SSFP imaging resulted in higher image quality with sharper delineation of aortic vessel walls at the proximal thoracic aorta, while respiratory-gated 3D-Dixon imaging was often accompanied by a slightly blurred delineation of vessel walls.

Sharper vessel wall delineation in 2D-SSFP imaging is most likely the reason for the observed higher precision of 2D-SSFP-derived diameter measurements at the level of the SOV compared to 3D-Dixon imaging. Reported differences in image quality between the two ECG-gated imaging techniques, in particular regarding vessel wall delineation, are likely explained by the fact that the 2D-SSFP sequence was acquired during breath-holding, while 3D-Dixon imaging was performed during free-breathing using navigator gating. The respiratory gating window of 5 mm resulted in a slightly blurred appearance, particularly of aortic segments close to the moving diaphragm such as the SOV. However, blurring had only mild effects on measurements precision at the SOV and no effect on measurements precision at all other aortic levels in respiratory-gated 3D-Dixon imaging, while the precision of aortic root measurements was without a significant difference compared to breath-hold 2D-SSFP imaging. Narrowing of the respiratory gating window might reduce blurring and thereby increase the precision of aortic measurements, however, at the cost of increased acquisition time. Future studies are needed to assess whether narrowing of the respiratory gating window statistically impacts measurement precision of aortic dilatation and more importantly, subsequent clinical management of Marfan patients.

Another reason for the observed higher precision of 2D-SSFP-derived aortic measurements might be the differential slice increment of 4 mm for 2D-SSFP imaging (−5 mm for gapless coverage of 9-mm thick slices) and 1 mm for 3D-Dixon imaging (−1 mm for gapless coverage of 2-mm slice thickness). Of note, both readers were free to choose appropriate slices displaying the maximum aortic profile from the parasagittal image stacks.

Regarding the higher slice increment for 2D-SSFP imaging, the slice displaying the maximal profile of the aorta stands out more from adjacent slices. Hence, both readers were more likely to choose the identical slice for aortic measurements when using 2D-SSFP imaging, while they might have chosen different slices when using 3D-Dixon imaging, thus resulting in a higher variance of determined diameters. For future studies, a direct comparison of the same reconstructed plane with identical in-plane resolution and slice thickness would be of great interest to compensate for possible deviations due to differences in resolution and slice thickness between 2D and 3D techniques.

Regarding accuracy, both 3D-Dixon-derived and 2D-SSFP-derived diameter measurements of the SOV revealed a strong correlation with echocardiographic measurements. Inner aortic diameter measurements derived from both 3D-Dixon imaging and 2D-SSFP imaging were about 1 mm larger than leading-edge echocardiographic measurements. The bias of about 1 mm between MR-derived inner-to-inner diameters and echocardiography-derived leading-edge diameters is likely explained by the different orientations of measurement planes. MRI measurements are derived from a parasagittal plane while echocardiography measurements are derived from a parasternal long axis view. This mean difference is in line with a recent study comparing inner 2D-SSFP-derived aortic diameter measurements with echocardiography-derived leading-edge measurements [12]. Similarly, Hoey et al reported that cine-MRI produces even higher measurements (+ 2.0 mm) than echocardiography measuring the cusp-commissure dimension on cross-sectional through-plane images [29].

Imaging artifacts were stronger in 2D-SFFP imaging as compared to 3D-Dixon imaging at all aortic levels. Recent studies have shown that artifacts in SSFP imaging increase with field strength (1.5 vs. 3 T). Our study at 3 T revealed almost no image artifacts in 3D-Dixon imaging in Marfan patients prior to aortic root surgery. Future studies are warranted to investigate the feasibility of 3D-Dixon imaging in post-surgical Marfan patients at 3 T.

Another important advantage of the 3D-Dixon imaging sequence is the inherent acquisition of a 3D data set allowing for secondary multiplanar reformations and consecutively exact orthogonal measurements of aortic dilatation. This aspect is of particular relevance for Marfan patients reaching the diameter threshold of 50 mm, indicating elective aortic surgery. The 3D-Dixon data set allows reformations for exact orthogonal measurements of the aorta allowing for the determination of the maximal aortic diameter of the oval-shaped aortic root.

In a consequence of this study, 3D-Dixon sequence was implemented next to our routinely performed 2D-SSFP sequence in our MR protocol for annual aortic monitoring of all Marfan patients. Future studies are warranted to investigate the difference in aortic diameters determined manually on parasagittal source images and maximal aortic diameters determined on multiplanar orthogonal reformations using semi-automatic software solutions.

It may be regarded as a limitation that aortic diameters were only determined in the para-sagittal planes of source images without using secondary multi-planar reformations. However, we aimed to minimize possible bias introduced by sub-optimal secondary reformations and subsequent measurement errors by using the identically orientated source images for both 3D-Dixon and 2D-SSFP measurements. Assessment of identical source images allowed for reliable assessment of only those differences that can be attributed to different imaging (3D-Dixon vs. 2D-SSFP) and triggering techniques (respiratory gating vs. breath-holding).

In summary, both respiratory-gated 3D-Dixon and breath-hold 2D-SSFP imaging allow for accurate and precise aortic diameter measurements. The shorter acquisition time for 2D-SSFP imaging is advantageous from a clinical perspective if reducing scan time is important. Nevertheless, we recommend 3D-Dixon imaging for monitoring of aortic diameter due to reduced image artifacts and the possibility of orthogonal multiplanar reformations of the aortic root.

## Abbreviations

MPR: Multiplanar reformations
SFFP MRI: Steady-state free precession MR imaging
SOV: Sinuses of Valsalva

## Declarations

### Guarantor

The scientific guarantor of this publication is Prof. Bannas.

### Statistics and biometry

Our co-author Susanne Sehner has statistical expertise and provided statistical advice.

### Methodology

• prospective

• observational

• performed at one institution
